# Toward Precision and Less Invasive Surgery in Thyroid, Breast, and Endocrine Diseases: Current Advances and Future Directions

**DOI:** 10.3390/medicina62102033

**Published:** 2026-10-20

**Authors:** Irida Pano, Francesk Mulita, Vasileios Leivaditis

**Affiliations:** 1 Department of Clinical Subject, University of Medicine Tirana, 1005 Tirana, Albania; 2 Second Department of Surgery, Medical School, Democritus University of Thrace, 68100 Alexandroupolis, Greece; 3 Department of Cardiothoracic and Vascular Surgery, Westpfalz Klinikum, 67655 Kaiserslautern, Germany

Surgery remains a cornerstone of treatment for many thyroid, breast, and other endocrine diseases, but its goals have evolved beyond effective disease removal alone. Contemporary surgical care increasingly seeks to balance oncological efficacy with preservation of organ function, reduction of morbidity, and optimization of functional, cosmetic, and patient-reported outcomes. Advances in molecular diagnostics, image-guided interventions, oncoplastic techniques, and robotic surgery have expanded the available therapeutic options and reinforced a central principle of modern surgical practice: treatment should be tailored not only to the disease, but also to the individual patient [1,2,3,4,5]. The contributions to this Special Issue reflect this transition across thyroid, breast, and adrenal surgery, while also identifying important areas in which stronger evidence is required.

This evolution is particularly evident in thyroid disease. Contemporary recommendations increasingly emphasize risk-adapted management, shared decision-making, active surveillance in appropriately selected patients, and less extensive surgery when oncologically suitable [1]. Diagnostic uncertainty nevertheless remains an important challenge, particularly for nodules with indeterminate cytology. Molecular testing can improve preoperative risk stratification and may reduce unnecessary diagnostic surgery, although its clinical utility depends on patient selection, disease prevalence, test characteristics, and availability [2]. Against this background, Türk et al. examined the role of repeat fine-needle aspiration biopsy after an initial diagnosis of atypia of undetermined significance (AUS) (Contribution 1). Their findings highlight the heterogeneity of malignancy risk within this cytological category and support a broader approach in which repeat cytology is interpreted alongside clinical and imaging characteristics rather than used as an isolated determinant of surgery. Future studies should evaluate integrated diagnostic pathways combining ultrasound, cytology, molecular testing, and patient preferences to determine which patients truly benefit from additional testing.

The changing boundary between surgical and non-surgical treatment is further illustrated by thyroid thermal ablation. Long-term evidence supports substantial and sustained volume reduction after thermal ablation of benign thyroid nodules, together with improvement in symptoms and cosmetic concerns [3]. Antzoulas et al. reviewed radiofrequency, microwave, and laser ablation, emphasizing their potential as thyroid-preserving alternatives to conventional surgery in appropriately selected patients in Contribution 2. These approaches are particularly relevant to the broader trend toward organ preservation, but important questions remain regarding patient selection, regrowth, repeat treatment, long-term surveillance, and standardization of techniques. Future comparative studies should therefore evaluate ablation against surgery and surveillance using not only technical efficacy, but also complications, thyroid function, quality of life, reintervention rates, and cost-effectiveness [3].

At the same time, refinement of conventional thyroid surgery remains essential. Post-thyroidectomy cervical hematoma is uncommon but potentially life-threatening, and established risk factors include patient-, disease-, and procedure-related characteristics such as male sex, older age, Graves’ disease, hypertension, antithrombotic therapy, previous thyroid surgery, bilateral procedures, and neck dissection [4]. The next paper published in this Special Issue adds an intriguing organizational dimension to this problem. Karabacak et al. identified hyperthyroidism and an operative start time at or after 15:00 as independent factors associated with postoperative cervical hematoma (Contribution 3). Although this single-center finding requires external validation, it raises the important possibility that operative scheduling and perioperative workflow may interact with conventional clinical risk factors. Larger multicenter studies could determine whether such potentially modifiable factors have reproducible effects on postoperative safety.

This Special Issue also reflects the continuing transformation of breast-conserving surgery. Oncoplastic techniques combine oncological resection with reconstructive principles and can facilitate breast conservation while maintaining favorable surgical and oncological outcomes. A recent meta-analysis comparing oncoplastic with conventional breast-conserving surgery reported lower rates of positive margins, re-excision, and subsequent mastectomy with oncoplastic approaches [5]. Within this evolving field, Mohamed et al. prospectively evaluated racquet mammoplasty for lateral-quadrant breast cancers (Contribution 4), reporting negative surgical margins in all included patients, a low complication rate, and favorable cosmetic outcomes. Abdelrahman et al. addressed a different anatomical setting by comparing matrix rotation with single-incision lateral sulcus mammoplasty for upper-quadrant tumors (Contribution 5), demonstrating differences in postoperative and esthetic outcomes between the two approaches. Together, these contributions illustrate an important principle of modern oncoplastic surgery: technique selection should reflect tumor location, breast anatomy, expected resection volume, and patient priorities rather than rely on a uniform operative strategy.

The evidence base for individual oncoplastic techniques, however, still requires maturation. Future studies should emphasize prospective multicenter comparisons, longer oncological follow-up, standardized assessment of complications and esthetic outcomes, and validated patient-reported measures. This is particularly relevant because the intended benefits of oncoplastic surgery extend beyond margin status to breast satisfaction, body image, psychosocial wellbeing, and quality of life. Systematic evidence using BREAST-Q supports the value of incorporating such patient-reported outcomes when evaluating breast-conserving and oncoplastic procedures [6].

Margin management in ductal carcinoma in situ (DCIS) represents another area in which individualized decision-making is increasingly important. Current consensus recommendations support a 2 mm negative margin for DCIS treated with breast-conserving surgery and whole-breast irradiation, while emphasizing that a negative margin narrower than 2 mm does not by itself mandate mastectomy and should be interpreted in the broader clinical context [7]. Karabacak et al. directly address this debate. In their retrospective cohort, they found that margins below 1 mm were associated with increased ipsilateral breast tumor recurrence, whereas the broader category of margins below 2 mm did not demonstrate uniform risk (Contribution 6). These findings do not replace established consensus recommendations, but they underscore why margin width should not be considered in isolation. Future studies should integrate margin status with patient age, lesion characteristics, adjuvant treatment, and other predictors of recurrence to refine individualized decisions regarding re-excision.

Preservation of form and quality of life is equally relevant in mastectomy and reconstruction. Skin- and nipple-sparing approaches can provide favorable reconstructive outcomes but are associated with specific risks, including skin-flap or nipple–areola complex necrosis, wound complications, infection, and implant loss [8]. Akyuz et al. examined these challenges in patients undergoing unilateral skin-sparing mastectomy with direct implant-based reconstruction (Contribution 7). Their study identified excision volume as a factor associated with wound complications and showed that several nipple–areola complex and wound complications could be managed with advanced wound care rather than reoperation. This contribution highlights the importance not only of preventing complications but also of developing effective strategies for reconstructive salvage when they occur. Future research should combine objective complication measures with patient-reported outcomes, since breast satisfaction, physical wellbeing, psychosocial function, and the burden of revision procedures are essential components of reconstructive success [9].

Technological innovation is also reshaping adrenal surgery. Comparative evidence indicates that robotic adrenalectomy is feasible and safe, with some perioperative advantages reported over conventional laparoscopy, although overall complication and readmission rates appear broadly comparable [10]. Ku et al. extend this discussion by comparing conventional laparoscopic adrenalectomy with single-port robotic adrenalectomy (Contribution 8). Their findings provide contemporary evidence regarding the perioperative performance of a newer robotic platform and illustrate a broader challenge in surgical innovation: technical feasibility alone is insufficient to establish clinical superiority. Future comparative studies should therefore examine complications, conversion, postoperative recovery, learning curves, patient-reported outcomes, and costs in addition to conventional measures such as operative time and blood loss [10].

Taken together, the eight contributions reveal several interconnected directions in contemporary endocrine and breast surgery. Türk et al. address more selective diagnostic decision-making before thyroid surgery (Contribution 1), while Antzoulas et al. explore non-surgical thyroid treatment and organ preservation (Contribution 2). Karabacak et al. examine potentially modifiable determinants of thyroidectomy safety (Contribution 3). The studies of Mohamed et al. and Abdelrahman et al. demonstrate how oncoplastic breast surgery is becoming increasingly adapted to tumor location and individual anatomy (Contributions 4 and 5), while the second study by Karabacak et al. revisits the balance between oncological safety and surgical extent in DCIS (Contribution 6). Akyuz et al. focus on preserving reconstructive outcomes when wound complications occur (Contribution 7), and Ku et al. examine the continuing technological evolution of minimally invasive adrenal surgery (Contribution 8). Thus, although the studies address different diseases and procedures, they converge on a common objective: achieving effective treatment while limiting unnecessary intervention and preserving outcomes that matter to patients.

This Special Issue also highlights important limitations in the current evidence base. Several questions surrounding novel procedures and refined surgical strategies continue to rely on single-center or retrospective data, while even prospective studies may have limited sample sizes or follow-up. Future research should therefore prioritize multicenter collaboration, standardized outcome definitions, prospective comparative designs, and sufficiently long follow-up, particularly when innovations are introduced into oncological practice. Technical endpoints should increasingly be complemented by functional outcomes, quality of life, patient satisfaction, and the need for subsequent interventions [6,9].

Ultimately, progress in thyroid, breast, and endocrine surgery is unlikely to be defined by a single technique or technology. For some patients, innovation may mean advanced oncoplastic or robotic surgery; for others, it may mean thermal ablation, a less extensive operation, surveillance, or avoidance of an unnecessary procedure. The contributions to this Special Issue collectively illustrate this transition from a predominantly procedure-centered model toward more selective, evidence-based, and patient-centered care. The next challenge is to validate promising approaches across broader populations and institutions and to determine not merely what is technically possible, but which intervention provides the greatest meaningful benefit for each patient.
